# Co_3_O_4_−CeO_2_ Nanocomposites for Low‐Temperature CO Oxidation

**DOI:** 10.1002/chem.202100927

**Published:** 2021-06-10

**Authors:** Jingxia Yang, Nevzat Yigit, Jury Möller, Günther Rupprechter

**Affiliations:** ^1^ Institute of Materials Chemistry Technische Universität Wien Getreidemarkt 9/BC/01 1060- Vienna Austria; ^2^ College of Chemistry and Chemical Engineering Shanghai University of Engineering Science Longteng Rd 333, Songjiang Shanghai, (P.R. China

**Keywords:** catalysis, CO oxidation, nanocomposites, solvothermal synthesis

## Abstract

In an effort to combine the favorable catalytic properties of Co_3_O_4_ and CeO_2_, nanocomposites with different phase distribution and Co_3_O_4_ loading were prepared and employed for CO oxidation. Synthesizing Co_3_O_4_‐modified CeO_2_ via three different sol‐gel based routes, each with 10.4 wt % Co_3_O_4_ loading, yielded three different nanocomposite morphologies: CeO_2_‐supported Co_3_O_4_ layers, intermixed oxides, and homogeneously dispersed Co. The reactivity of the resulting surface oxygen species towards CO were examined by temperature programmed reduction (CO‐TPR) and flow reactor kinetic tests. The first morphology exhibited the best performance due to its active Co_3_O_4_ surface layer, reducing the light‐off temperature of CeO_2_ by about 200 °C. In contrast, intermixed oxides and Co‐doped CeO_2_ suffered from lower dispersion and organic residues, respectively. The performance of Co_3_O_4_‐CeO_2_ nanocomposites was optimized by varying the Co_3_O_4_ loading, characterized by X‐ray diffraction (XRD) and N_2_ sorption (BET). The 16–65 wt % Co_3_O_4_−CeO_2_ catalysts approached the conversion of 1 wt % Pt/CeO_2_, rendering them interesting candidates for low‐temperature CO oxidation.

## Graphical abstract

**Co_3_O_4_−CeO_2_ nanocomposites** with three different morphologies, that is, CeO_2_‐supported Co_3_O_4_ layers, intermixed oxides, and homogeneously dispersed Co, were used for CO oxidation. Both CO‐TPR and CO oxidation kinetics indicated that the layered structure exhibited the best performance for the same Co_3_O_4_ loading of 10.4 wt %. The CO oxidation activity increased for higher Co_3_O_4_ amount, approaching activity comparable to Pt/CeO_2_.

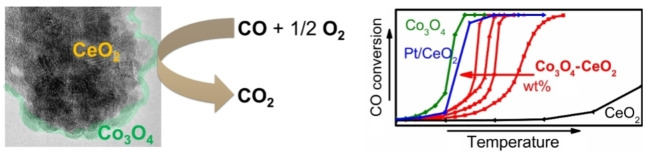

## Introduction

Ceria (CeO_2_) has applications in many catalytic reactions, such as methane dry reforming,^[1]^ hydrocarbon and diesel soot oxidation,^[2]^ organic synthesis^[3]^ and especially environmental catalysis.^[4]^ Ceria is a crucial component of three‐way‐catalysts (TWCs), serving together with alumina as support for dispersed noble metal (e. g., Pt (or Pd) and Rh) nanoparticles, preventing their sintering at high temperatures. Additionally, ceria regulates the surface oxygen concentration under fuel lean and rich conditions, due to its oxygen buffer (storage/release) capacity (OBC/OSC), associated with the fast Ce^4+^/Ce^3+^ redox cycle. Recently, advanced synthesis methods allowed to develop nanostructured ceria of various morphologies (shapes), with high specific surface area (SSA) and improved Ce^3+^/Ce^4+^ ratio.[^4a^, ^4d^, ^5^] Furthermore, noble metals supported on ceria exhibit remarkable catalytic activity in preferential CO oxidation (PROX)^[6]^ and water gas shift (WGS),^[7]^ attributed to active sites at the metal/support interface^[8]^ and the availability of lattice oxygen (oxygen vacancies).^[9]^ CeO_2_ is thus considered an active (“non‐innocent”)^[10]^ support.

Increasingly stringent emission regulations require continuous innovations, especially regarding engine cold start emissions at temperatures when noble metals are CO‐poisoned and inactive.^[11]^ For noble metals, temperatures around 100–200 °C are typically required to initiate CO oxidation (“ignition”),[^11a^, ^12^] which is why ∼80 % of the emissions result from the cold‐start period. Therefore, many studies focused on low‐temperature CO oxidation, for example, over Au nanoparticles (2–4 nm size) on reducible oxides (e. g., TiO_2_, CeO_2_). These are less prone to CO poisoning, but Au nanoparticles tend to sinter. Co_3_O_4_ has been shown to be very active,[^4b^, ^13^] but fully replacing CeO_2_ by Co_3_O_4_ would require substantial modifications of the existing TWC technology, as the CeO_2_ support is important for both oxidation and reduction.^[14]^ However, Co_3_O_4_‐modified CeO_2_ nanocomposites may be a compromise,^[15]^ combining the favorable activity of cobalt oxide with the high oxygen storage capacity of ceria. Most importantly, for such mixed or supported oxides the limitations of availability and costs of noble metals apparently do not apply.

However, apart from the mere basic composition, the performance of Co_3_O_4_−CeO_2_ catalysts strongly depends on the applied synthesis route, which determines the dispersion, morphology/microstructure, surface composition, redox and catalytic properties of the oxides. The materials reported so far in the literature were mainly prepared by conventional impregnation and coprecipitation methods followed by high temperature calcination, which does not yield catalysts with the desired high surface area and high dispersion of oxide phases, which are prerequisites for high catalytic activity in CO oxidation.^[16]^

Previously, we have presented a new feasible route for preparation of Co_3_O_4_‐modified CeO_2_ catalysts, based on sol‐gel synthesis combined with solvothermal processing, permitting the direct crystallization of the gel without the need of annealing at high temperatures to induce crystallization.^[17]^ Using this approach, we have been able to synthesize Co_3_O_4_‐modified CeO_2_ nanocomposites with high specific surface area and highly dispersed cobalt oxide nanoparticles. This maximizes the number of accessible active sites and results in high CO oxidation activity of Co_3_O_4_‐modified CeO_2_ catalysts, comparable to that of pure Co_3_O_4_ and, in terms of activation energy, even to Pt/ and Pd/CeO_2_.^[18]^ Au/CeO_2_ shows activity at lower temperature, due to lower CO binding energy,[^4e^, ^7d^, ^19^] but a coinage metal is apparently required.

Based on the effect of different synthesis routes on the structure of Co_3_O_4_‐modified CeO_2_ reported previously, herein the available oxygen species, redox properties and catalytic performance are examined in detail. Furthermore, an optimum Co_3_O_4_ loading of CeO_2_ is determined. Pure CeO_2_ and Co_3_O_4_, as well as Pt/CeO_2_, are included for comparison. The Co_3_O_4_‐CeO_2_ nanocomposites turned out to be promising candidates for low‐temperature CO oxidation and could be implemented by moderate modifications of TWC manufacture.

## Results and Discussion

Table 1 summarizes the three synthesis routes and the different resulting structures of the Co_3_O_4_ modified CeO_2_ catalysts, as reported previously.^[17]^ Herein, the focus is on their reduction by CO, reflecting the active oxygen species and ability for oxygen vacancy formation, which are important for the catalytic flow reactor performance. Then, the Co_3_O_4_ loading is tuned, including further characterization and activity measurements. The catalytic performance of the Co_3_O_4_‐CeO_2_ nanocomposites is finally contrasted to those of the pure oxides and Pt/CeO_2_.

**Table 1:** Overview of three different sol‐gel routes for synthesis of Co_3_O_4_‐CeO_2_ nanocomposites and of corresponding structures (details please see the Supporting Information).

Route	Procedure	Structure^[17]^
**1**	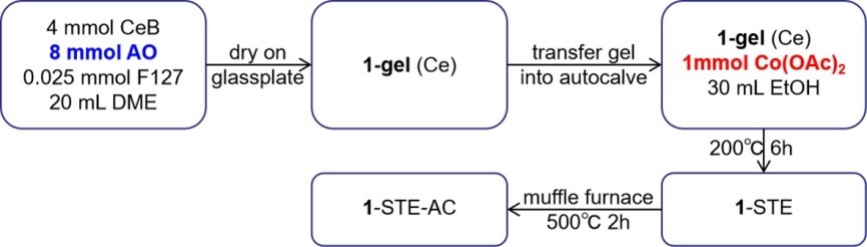	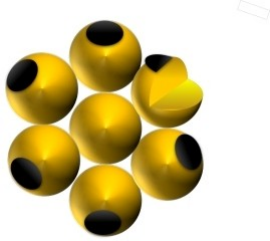
		small (3–5 nm) Co_3_O_4_ particle aggregation (black), forming layers on the surface of larger (10–20 nm) agglomerated CeO_2_ particles (yellow): “supported Co_3_O_4_”
**2**	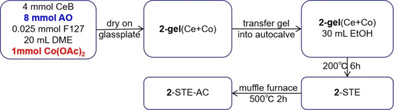	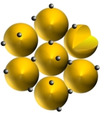
		coexisting (3–5 nm) Co_3_O_4_ (grey) and (20–30 nm) CeO_2_ nanoparticles (yellow): “intermixed oxides”
**3**	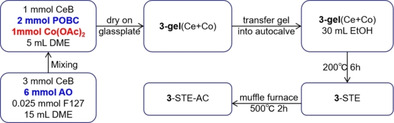	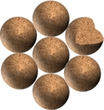
		homogeneous distribution of cobalt within CeO_2_ (via Co‐O−Ce network): “Co‐doped CeO_2_”

### CO‐TPR of pure CeO_2_ and Co_3_O_4_

**Figure 1:**
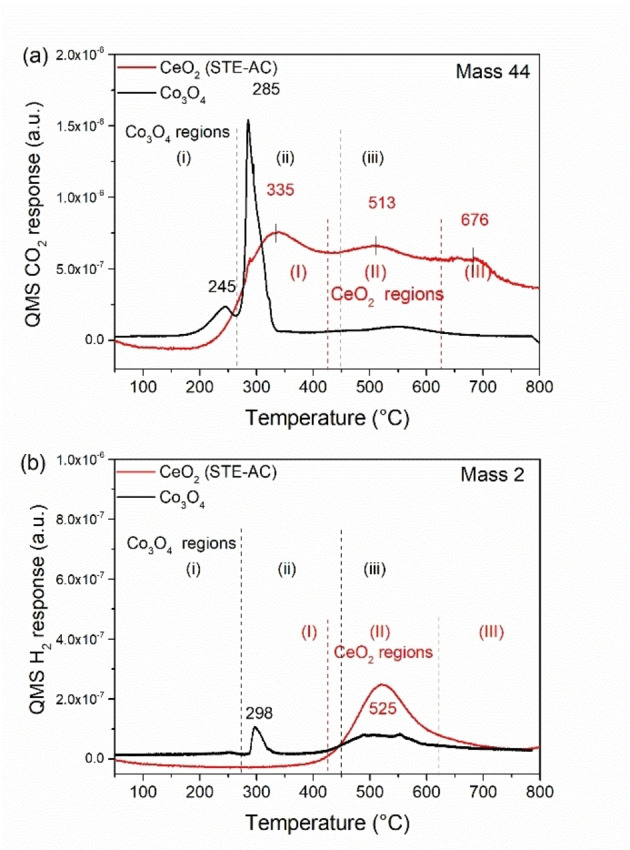
CO‐TPR of pure CeO_2_ (STE‐AC) and commercial Co_3_O_4_: (a) CO_2_ and (b) H_2_ evolution.

First, the pure oxides were characterized by CO temperature programmed reduction, with the results being in line with the literature.[^4d^, ^15a^, ^20^] For pure CeO_2_, prepared by the combination of sol‐gel and solvothermal methods in ethanol (STE), and further air calcined (AC) at 500 °C (i. e., STE‐AC),^[4d]^ the CO_2_ evolution in CO‐TPR occurs in three regions (Figure 1a, red line): (I) 250–425 °C, due to removal of surface lattice oxygen (O_SL_); CO+O_SL_→CO_2_; (II) 425–625 °C, due to water gas shift (WGS) between CO and surface OH groups (CO+OH→CO_2_ +1/2 H_2_); (III)>625 °C, due to extraction of bulk lattice oxygen (O_BL_). Accordingly, due to WGS the H_2_ evolution on CeO_2_ exhibits a main peak in region II (Figure 1b). Still, the reducibility of ceria is much lower than that of Co_3_O_4_.

Co_3_O_4_ is reduced at lower temperature and several studies^[20]^ reported a two‐step process: Co^3+^→Co^2+^→Co^0^. This was confirmed by CO‐TPR of commercial Co_3_O_4_, as evident from Figure 1 (black lines). The CO_2_ evolution (Figure 1a) occurs in three stages: (i) <270 °C, via O_SL_ removal; CO+O_SL_→CO_2_; Co^3+^_SL_→Co^2+^_SL_; (ii) 270–450 °C, O_BL_ removal of bulk lattice oxygen (CO+O_BL_→CO_2_), causing Co^3+^_BL_→Co^2+^_BL_, and further reduction of Co^2+^→Co^0^.^[21]^ In the 450–670 °C region, CO disproportionation (2CO ***→*** CO_2_+C) or CO dissociation (CO ***→*** C+O) may occur on Co^0^, resulting in minute CO_2_ desorption.^[22]^ Additionally, some H_2_ evolution (Figure 1b) is detected around 300 °C, pointing to WGS of CO with surface OH groups.^[23]^ Some OH groups only react around 500 °C. Both for CeO_2_ and Co_3_O_4_ mass 18 was also recorded, but no water desorbed over the entire temperature range. CO‐TPR spectra (mass 44 and 2) of a 1 : 1 physical mixture of CeO_2_/Co_3_O_4_ were simply a superposition of the spectra of the individual oxides, indicating that oxide/oxide interactions were absent in this case.

### CO‐TPR of 10 wt % Co_3_O_4_‐modified CeO_2_ synthesized via routes 1–3

For Co_3_O_4_–modified CeO_2_ different and more complex CO‐and H_2_‐TPR profiles were obtained, that were not just a sum of the profiles of the individual oxides. First, new low temperature peaks indicate additional surface oxygen species and, second, the “individual oxide peaks” are shifted by 20 or more degrees to higher temperature. Both indicate synergetic interfacial interactions between cobalt oxide and ceria, affecting the Co^3+^/Co^2+^ and Ce^4+^/Ce^3+^ redox properties, which seems to promote the reactive oxygen species. This also holds true for H_2_ evolution.[^20b^, ^24^]

**Figure 2:**
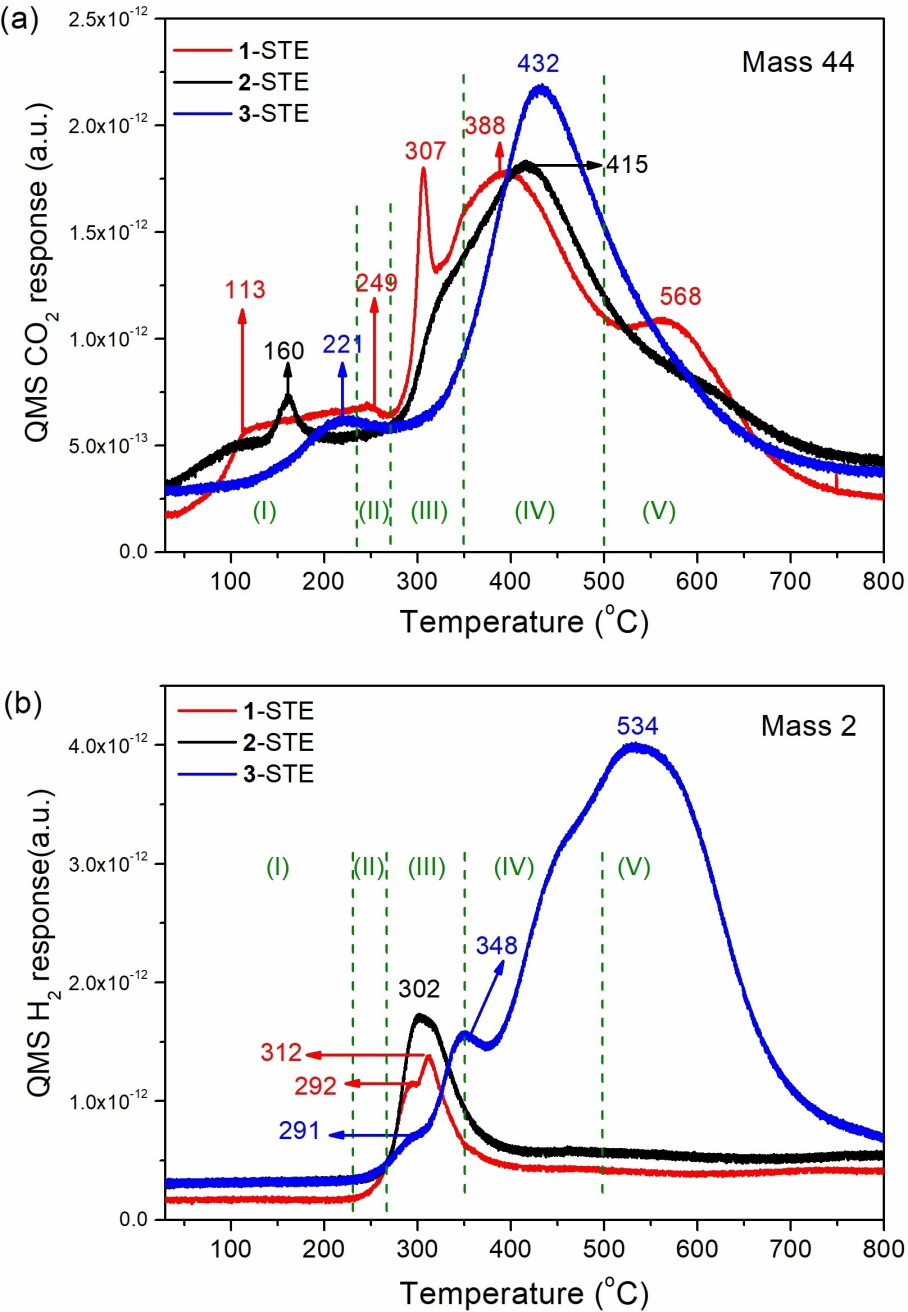
CO‐TPR of 10.4 wt. % Co_3_O_4_ modified CeO_2_ STE samples: (a) CO_2_ and (b) H_2_ evolution.

**Figure 3:**
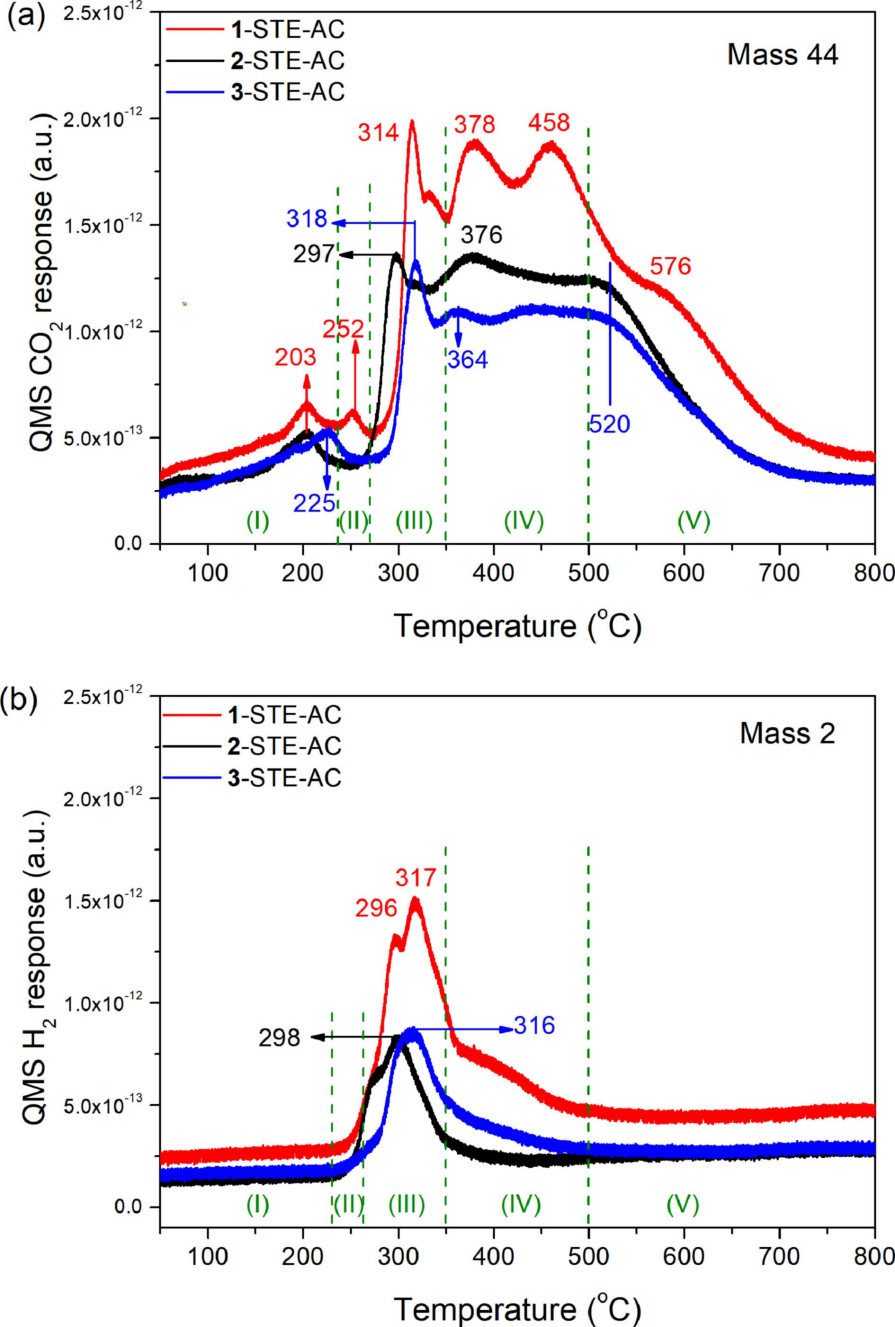
CO‐TPR of 10.4 wt. % Co_3_O_4_ modified CeO_2_ STE‐AC samples: (a) CO_2_ and (b) H_2_ evolution.

Accordingly, CO‐TPR was performed for all six samples (three *STE* (Figure 2) and the corresponding *STE‐AC* (Figure 3) samples). The different profiles clearly show that the interaction with CO/reducibility (CO_2_ evolution) strongly depends on the preparation route and the heat‐treatment (calcination), as both affect the Co distribution. Based on the CO‐TPR results in Figure 1 and the literature,[^20c^, ^24a^, ^24c^, ^25^] the peaks of all synthesized nanocomposites, except from **3**‐STE, can be assigned to five regions:

1. Region I (<240 °C): Reaction of CO with reactive oxygen species, very likely on Co_3_O_4_, present after the oxidative pretreatment. Molecularly adsorbed oxygen on Co^2+^ dissociates to atomic oxygen (O−), transforming Co^2+^ to Co^3+^. CO may also react with this oxygen species to carbonates, that decompose below 200 °C.^[4b]^
2. Region II (240‐270 °C): Reaction of O_SL_ at Co_3_O_4_/CeO_2_ interfaces and reduction of Co^3+^→Co^2+^: CO+O_SLCo3O4_ →CO_2_;
3. Region III (270‐350 °C): Reaction of O_BL_ near Co_3_O_4_/CeO_2_ interfaces: CO+O_BLCo3O4_→CO_2_; Co^3+^→Co^2+^. Reaction of O_BL_ of CoO, interacting with CeO_2_, producing Co^0^: CO+O_BLCoO_ → CO_2_; Co^2+^→Co^0^. WGS reaction of OH on Co_3_O_4_, including both isolated Co_3_O_4_ (at slightly lower temperature) and the Co_3_O_4_/CeO_2_ interface (at slightly higher temperature): CO+OH→1/2 H_2_+CO_2_;
4. Region IV (350–500 °C): removal of O_SL_ of CeO_2_ interacting with Co_3_O_4_; CO+O_SL_→CO_2_;
5. Region V (>500 °C): Reaction of O_BL_ of CeO_2_: CO+O_BLCeO2_→CO_2_; Ce^4+^→Ce^3+^.

The CO_2_ evolution in region III is related to Co_3_O_4_ surface patches, from which the surface abundance of the modified Co_3_O_4_ phase can be deduced. For STE samples, only sample **1**‐STE has a sharp peak in this region (Figure 2a), while sample **2**‐STE has a weak shoulder and sample **3**‐STE has no peak. This indicates that the amount of the available Co_3_O_4_ on or near the ceria surface is **1**‐STE>**2**‐STE>**3**‐STE (in line with HRTEM results).^[17]^ For sample **3**‐STE, the intense peak of CO_2_ evolution (432 °C: Figure 2a) is caused by the decomposition of the POBC ligand, which is still present after the solvothermal treatment. This is reflected in the lower temperature shoulder of the 534 °C peak of H_2_ evolution (Figure 2b). The latter peak and the one at 348 °C once more indicate WGS of CO with surface OH groups.

To examine how high temperature calcination influences reactivity/reducibility, the STE samples were air‐calcined at 500 °C for 2 h (STE‐AC). After calcination, all STE‐AC samples showed low temperature peaks in region I, and especially sharp peaks in region III. The different intensities in region III indicate that part of the Co in sample **2**‐STE‐AC and **3**‐STE‐AC sintered and/or segregated to the surface (Figure 3). The higher temperature peaks are related to ceria. Once more, the intensity of the peaks in regions I–III indicates the amount of Co oxide that is available for CO oxidation. Compared to pure Co_3_O_4_ (Figure 1) and uncalcined nanocomposites (Figure 2), the peaks in region III are even more shifted to higher temperature, indicating an increased interaction with CeO_2_. For **2**‐STE‐AC and **3**‐STE‐AC, though some Co aggregated, some Co is still in the bulk of CeO_2_, and may thus not participate in CO oxidation. The H_2_ evolution of STE‐AC samples in region III (Figure 3b), due to WGS, showed a similar trend. Sample **1**‐STE‐AC formed the most H_2_, while **2**‐STE‐AC and **3**‐STE‐AC produced only half the H_2_ amount. Thus, it can be deduced that **1**‐STE‐AC exhibited most active Co_3_O_4_ interacting with CeO_2_, while the other two samples had only about half the amount of available CoO_x_/CeO_2_. This is consistent with high‐resolution transmission electron microscopy (HR‐TEM) micrographs of the AC samples.^[17]^

### Catalytic performance of 10 wt % Co_3_O_4_‐modified CeO_2_ synthesized via routes 1–3

**Figure 4:**
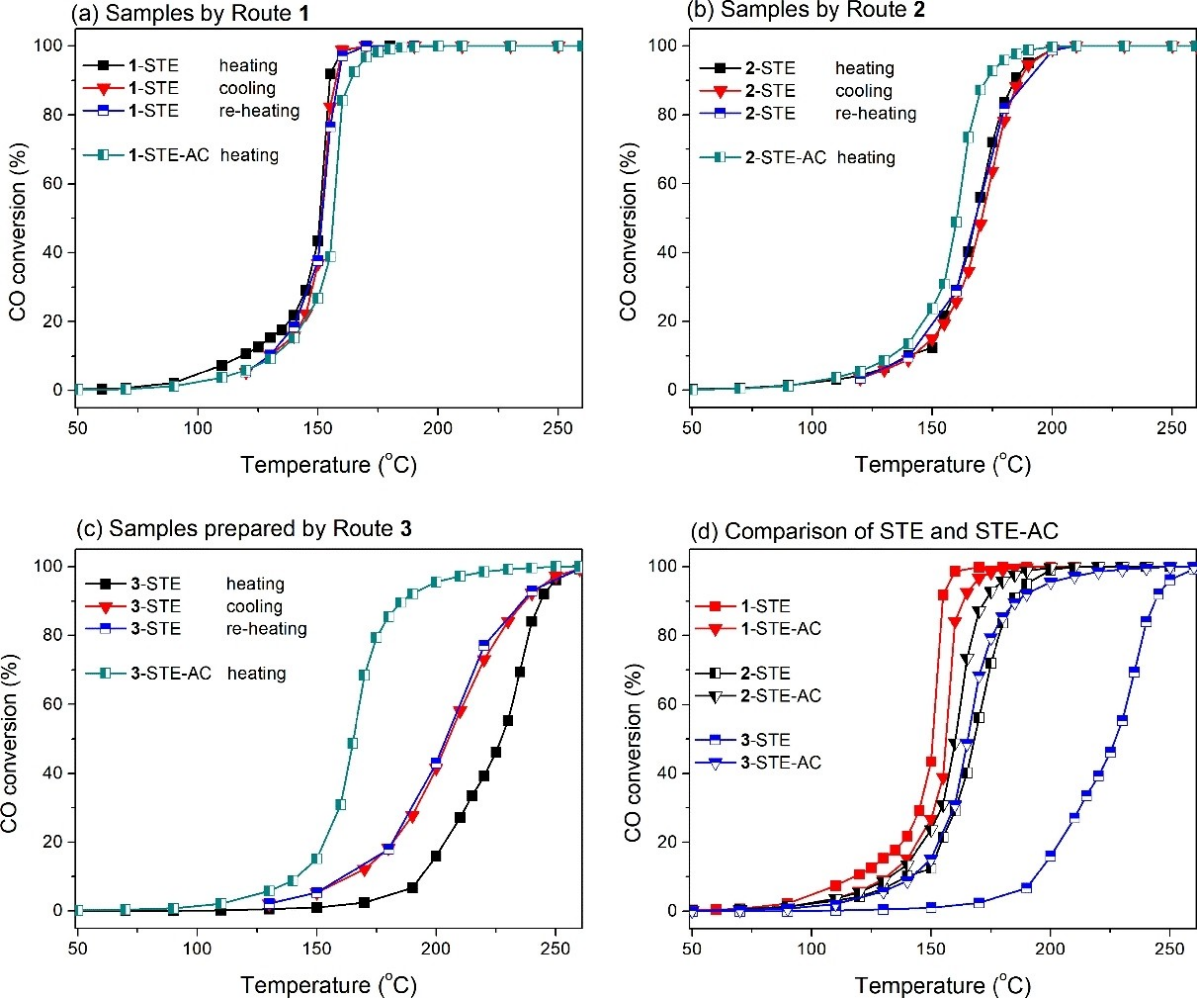
CO oxidation over 10.4 wt % Co_3_O_4_ modified CeO_2_ samples. (a) route 1, (b) route 2, (c) route 3 and (d) direct comparison of 1^st^ heat‐up.

The catalytic activity of the six samples (three *STE* samples by different routes and the corresponding *STE‐AC* samples) in CO oxidation (5 % CO, 10 % O_2_, He balance) was evaluated at different temperatures (Figure 4). Trends were tabulated previously;^[17]^ herein, more detailed catalytic tests are contrasted to the characterization discussed above. For practical applications, apart from the catalytic activity, the thermal stability of catalysts is most crucial. To detect a potential loss of catalytic activity, the CO conversion was thus recorded upon heating, upon subsequent cooling and upon re‐heating, *without* intermittent catalyst reactivation. Catalysts that underwent such cycling are stable in isothermal reactions up to 200 °C, typically over hundreds of hours.

Samples **1**‐STE (Figure 4a) and **2**‐STE (Figure 4b) (prepared by two individual precursors) did not show any hysteresis or loss of activity within several test cycles. In contrast, for sample **3**‐STE (Figure 4c) the CO conversion upon heating and subsequent cooling do not coincide anymore. Interestingly, the CO conversion at a given temperature became apparently higher upon cooling and then remained the same during re‐heating. This behavior of **3**‐STE (for which a single source precursor was used) can be explained by the decomposition of residual organics (e. g., POBC ligand) upon heating to ∼250 °C in oxidative atmosphere (which is supported by previous thermogravimetric analysis (TGA)).^[17]^ The highest activity (at a given temperature) of the catalyst prepared by route **1** can be explained by the more active and more abundant Co_3_O_4_ on the CeO_2_ surface (layer structure).^[17]^

In order to determine the effect of calcination, the *STE‐AC* samples were also tested in CO oxidation (Figure 4). **1**‐STE‐AC exhibited somewhat lower CO conversion than its STE pendant (Figure 4a). In contrast, when comparing the other STE samples with the corresponding STE‐AC samples (Figure 4b, c), at a given temperature **2**‐STE‐AC and especially **3**‐STE‐AC had a higher CO conversion than the related STE samples. Apparently, the significant amount of organic residues, which had remained after solvothermal treatment, blocked reaction sites, resulting in lower activity. Thus, the high temperature calcination is beneficial for **2**‐STE and **3**‐STE to remove organic residues. Nevertheless, the high temperature calcination also caused a structure collapse and reduction in surface area (from 216∼217 m^2^ g^−1^ to 25∼96 m^2^ g^−1^),^[17]^ which is why annealing of **1**‐STE (with the smallest amount of organic residues) resulted in somewhat lower catalytic activity of **1**‐STE‐AC. Therefore, for **1**‐STE the post‐synthesis calcination should be avoided. The performance of all catalysts in the first heating cycle is directly compared in Figure 4d.

**Figure 5:**
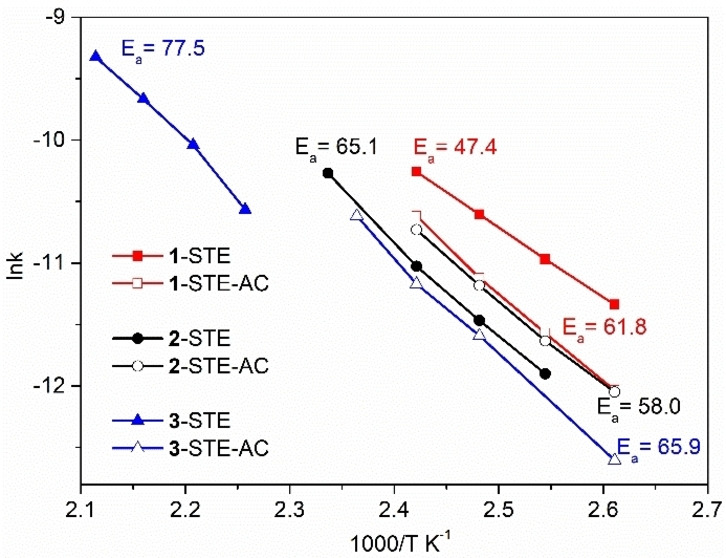
Arrhenius‐type plots for CO oxidation over STE and STE‐AC samples (error: ±2 kJ mol^−1^).

The apparent activation energy (*E_a_*) of all STE and STE‐AC samples was calculated from Arrhenius‐type plots using kinetic rates below 30 % conversion (Figure 5). **1**‐STE has the lowest *E_a_* of 47.4 kJ mol^−1^, while 3‐STE shows the highest *E_a_* of 77.5 kJ mol^−1^. The *E_a_* of **1**‐STE is similar to that of noble metals supported on ceria, such as 0.5 wt % Pd/CeO_2_ (48–52 kJ mol^−1^,^[9b]^ 1 wt % Pd/CeO_2_ (40 kJ mol^−1^)),^[26]^ 0.5 wt % and 1 wt % Pt/CeO_2_ (42–63^[9b]^ and 44 kJ mol^−1^,^[27]^ respectively) and Au/CeO_2_ (46–56 kJ mol^−1^).^[28]^ Thus, the combination of sol‐gel and solvothermal methods allows obtaining very active cobalt oxide‐modified ceria nanocomposites, which could be used as a low‐temperature‐active additive to noble metal loaded CeO_2_.

The promising activity of 10.4 wt % Co_3_O_4_−CeO_2_ catalysts (route 1) is attributed to the favorable phase distribution, i. e., a high dispersion and small crystallite size of the active Co_3_O_4_ phase on CeO_2_. ^[29]^ The oxygen availability seems promoted by synergetic interfacial interactions between cobalt and ceria (Co‐O−Ce bonds), modifying the Co^2+^/Co^3+^ and Ce^3+^/Ce^4+^ redox properties and producing more active oxygen species.[^20b^, ^30^] Along these lines, the Co_3_O_4_ loading on CeO_2_ catalysts was varied, as described in the following.

### Optimizing the Co_3_O_4_ loading on CeO_2_ (via route 1)

To further improve the performance of Co_3_O_4_‐modified CeO_2_, the number of Co_3_O_4_ surface sites accessible to the reaction was optimized via the Co_3_O_4_ loading. As route **1** produced the material with the highest CO oxidation activity, additional catalysts were prepared following this route, but with different amounts of cobalt precursor. The molar percentage of Co(Oac)_2_/(CeB+Co(Oac)_2_) used for all samples was 10, 20, 30 and 80 %, translating to Co_3_O_4_/(Co_3_O_4_+CeO_2_) wt. % ratios of 4.9, 10.4, 16.6 and 65.1 wt %, respectively.

**Figure 6:**
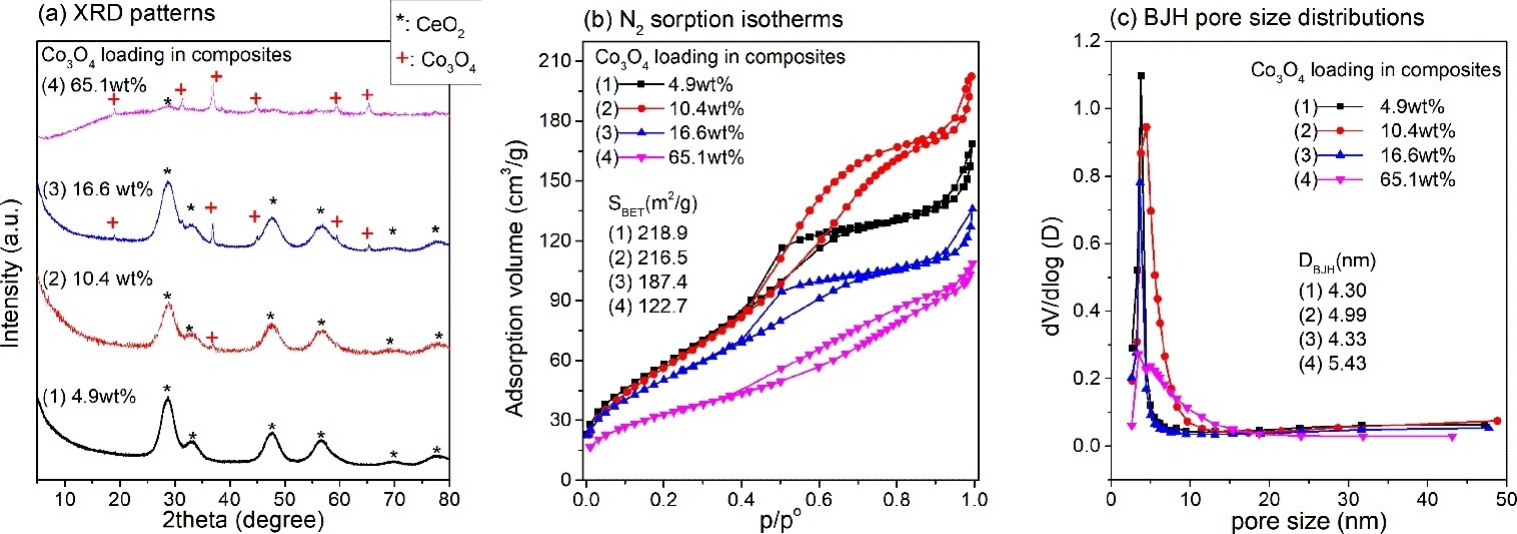
(a) XRD patterns, (b) N_2_ adsorption–desorption isotherms, and (c) pore size distributions of Co_3_O_4_‐CeO_2_ nanocomposites (1‐STE). The data of the 10.4 wt % sample were adapted with permission from ref. [17]. Copyright 2015, Wiley.

The 1‐STE samples with different Co_3_O_4_ loading were characterized by X‐ray diffraction (XRD) and N_2_ sorption (Figure 6). The XRD of 4.9 wt % Co_3_O_4_/CeO_2_ showed only diffraction peaks of CeO_2_, as Co was highly dispersed.^[31]^ The higher loadings exhibited features characteristic of both Co_3_O_4_ and CeO_2_, with the intensity of the Co_3_O_4_ diffraction peaks increasing with Co loading. The CeO_2_ crystals were in the size range of 2.5–3.5 nm, whereas that of Co_3_O_4_ was about 25 nm (similar to commercial Co_3_O_4_).

The textural properties of the differently loaded **1**‐STE catalysts were characterized by N_2_ adsorption. Adsorption‐desorption isotherms, the resulting specific surface area (SSA) and the pore size distributions are plotted in Figure 6 b,c. The specific surface area (S_BET_) decreased with increasing loading. While S_BET_ was about 220 m^2^/g for 4.9 wt % Co_3_O_4_/CeO_2_, it was only ∼120 m^2^/g for 65.1 wt % Co_3_O_4_/CeO_2_. Nevertheless, this is still large compared to the SSA of commercial Co_3_O_4_ (37 m^2^ g^−1^). Up to 16.6 wt % Co_3_O_4_/CeO_2_, the isotherms indicate mainly mesopores and a small proportion of macropores, with narrow pore size distributions. In contrast, the 65.1 wt % Co_3_O_4_/CeO_2_ sample is mostly macroporous with a small amount of mesopores. Thus, a suitable ratio between Co_3_O_4_ and CeO_2_ is crucial to preserve mesoporosity and to obtain a high specific surface area with a high dispersion of Co_3_O_4_ on CeO_2_. The structural data are summarized in Table 2.

**Table 2:** Crystallite size (P), specific surface area (S), and activity data for Co_3_O_4_‐modified CeO_2_ (1‐STE), pure CeO_2_ and Co_3_O_4_, as well as 1 wt % Pt/CeO_2_.

Co_3_O_4_ loading [wt %]	P_CeO2_^[a]^ [nm]	P_Co3O4_^[b]^ [nm]	S_BET_^[c]^ [m^2^ g^−1^]	T_10%_^[d]^ [°C]	T_90%_^[e]^ [°C]	r_100 °C_^[f]^ [mol s^−1^ g^−1^]	R_100 °C_^[g]^ [mol s^−1^ m^−2^]	R_Co100 °C_^[h]^ (mmol CO/mmol Co h^−1^]
**0 (Ceria)**	<3	/	277.0	253	398	/	/	/
**4.9**	3.0	/	218.9	138	197	3.90×10^−6^	1.78×10^−8^	22.9
**10.4**	3.5	/	216.5	117	155	7.78×10^−6^	3.59×10^−8^	21.6
**16.6**	2.5	27.5	187.4	105	148	1.23×10^−5^	6.56×10^−8^	21.4
**65.1**	3.3	23.7	122.7	105	134	1.21×10^−5^	9.87×10^−8^	7.4
**100 (Co_3_O_4_)**	/	28	37	84	114	4.14×10^−5^	1.12×10^−6^	11.9
**0 (Pt/CeO_2_)**	13.5	/	43.2	100	124	1.15×10^−5^	2.68×10^−7^	/

[a] CeO_2_ crystal particle size calculated by Scherrer equation from XRD (JCPDS card number of CeO_2_: 34–0394) [b] Co_3_O_4_ crystal particle size calculated by Scherrer equation from XRD (JCPDS card number of Co_3_O_4_: 42–1467) [c] BET surface area from N_2_ sorption [d] Reaction temperature for 10 % CO conversion [e] Reaction temperature for 90 % CO conversion [f] Reaction rate of CO oxidation at 100 °C per gram [g] Normalized specific reaction rates of CO oxidation on a unit surface area at 100 °C [h] Reaction rates per unit amount of Co at 100 °C.

### Catalytic performance of different Co_3_O_4_ loadings on CeO_2_ (via route 1)

**Figure 7:**
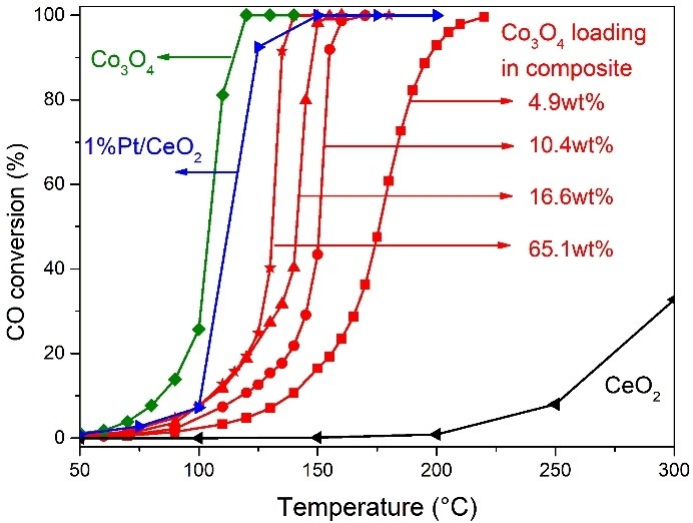
CO conversion vs. temperature of Co_3_O_4_‐modified CeO_2_ (STE) for different loadings. CeO_2_, Co_3_O_4_, and Pt/CeO_2_ serve as reference. Conditions: 5 % CO, 10 % O_2_, He balance; 10 mg catalyst.

The different **1**‐STE nanocomposites (without air‐calcination) were subsequently tested in CO oxidation, and contrasted to pure CeO_2_, pure Co_3_O_4_ and 1 wt. % Pt/CeO_2_ (the latter with a mean Pt particle size of 1.7 nm, according to CO chemisorption). Figure 7 shows the temperature‐dependent CO conversion of the different pretreated **1**‐STE samples and pretreated reference catalysts (10 mg each). The temperatures of 10 % CO conversion (T_10%_) for **1** *STE* samples are: 138 °C (4.9 wt %) >117 °C (10.4 wt %) >105 °C (16.6 wt %)=105 °C (65.1 wt %) >100 °C (1 wt % Pt/CeO_2_)>84 °C (Co_3_O_4_); the temperatures of 90 % CO conversion T(_90%_) are: 197 °C (4.9 wt %) >155 °C (10.4 wt %) >148 °C (16.6 wt %) >134 °C (65.1 wt %) >124 °C (1 wt % Pt/CeO_2_) >114 °C (Co_3_O_4_).

Apparently, pure CeO_2_ is the least active (30 % conversion at 300 °C), but already adding ∼4.9 wt. % Co_3_O_4_ drastically increased activity. This trend continued for 10.4, 16.6, and 65.1 wt %, the latter approaching the activity of Pt/CeO_2_. Clearly, the higher the Co percentage in the synthesis and thus the final Co_3_O_4_ loading are, the higher the resulting catalytic activity is. Pure Co_3_O_4_ is the most active, but for possible TWC‐applications, ∼16‐65 wt % Co_3_O_4_‐modified CeO_2_‐based catalysts seem the best. This agrees with a similar higher activity of Co_3_O_4_ catalysts impregnated by 10 wt. % CeO_2_ in preferential CO oxidation.^[15a]^

To compare the different samples, the catalytic activity/rate of the catalysts at 100 °C was normalized by weight (r_100 °C_), by specific surface area (R_100 °C_) and by the unit amount of Co (R_Co100 °C_), as listed in Table 2 (the CO conversion at 100 °C was below 20 % for all samples). Up to 16.6 wt % Co_3_O_4_, the values of r_100 °C_ and R_100 °C_ increased almost in direct proportion to the Co_3_O_4_ loading. For example, the r_100 °C_ and R_100 °C_ values of 4.9 % Co_3_O_4_ are 3.90×10^−6^ mol s^−1^ g^−1^ and 1.78×10^−8^ mol s^−1^ m^−2^, respectively. The corresponding values of 10.4 % Co_3_O_4_ are 7.78×10^−6^ mol/s g and 3.59×10^−8^ mol/s m^2^, i. e., each almost exactly double.

This is consistent with the R_Co100 °C_ values, as up to 16.6 wt % Co_3_O_4_ the samples have almost the same R_Co100 °C_ value of 22±1 mmol CO/mmol Co⋅h (Table 2). This indicates that Co_3_O_4_ is well dispersed, forming increasingly larger islands on the CeO_2_ surface. Further increasing the Co_3_O_4_ loading to 65 wt % hardly increased the specific activity and even decreased the rate normalized by the Co amount to 7.4 mmol CO/mmol Co⋅h, as agglomerated particles or thicker layers have less dispersion. This is clearly evident from the low temperature range (Figure 7), with the conversion of the 16.6 and 65 wt % samples being almost the same. Pure Co_3_O_4_ nanoparticles are characterized by a similar value (11.9 mmol CO/mmolCo⋅h), as the Co_3_O_4_ dispersion is was lower than that of thin Co_3_O_4_ layers.

Even though Co_3_O_4_ is known for high CO oxidation activity at low temperature, the use of pure Co_3_O_4_ in catalytic convertors is not feasible, as ceria has oxygen storage/release (buffer) capacity, in addition to preventing sintering. It is thus important to preserve the main CeO_2_ phase, but its low‐temperature activity could be boosted by well‐dispersed Co_3_O_4_ overlayers. As modern engines run under oxygen‐rich conditions to increase mileage, the exhaust gas is oxygen rich too, which stabilizes the Co_3_O_4_ phase, so that no reduction would occur (deactivation by adsorbed water may be a problem at lowest temperatures, though). The re‐oxidation of (metallic) cobalt starts above 200 °C, at 300 °C it is oxidized to CoO and Co_3_O_4_, and at 400 °C Co_3_O_4_ is the most stable phase.^[13b]^ Clearly, operando examination of the nanocomposites would add to the mechanistic understanding^[32]^ and Co_3_O_4_ may also be beneficial for NO_x_ reduction.^[33]^

## Conclusions

The current results demonstrate that the sol‐gel synthesis of Co_3_O_4_−CeO_2_ nanocomposites, in combination with solvothermal treatment, allows obtaining well‐dispersed cobalt oxide nanoparticles on high surface area CeO_2_. The obtained materials are thermally more stable and very active in CO oxidation, and are comparable to supported Pt−, Pd− and Au−CeO_2_ catalysts. The catalytic performance of Co_3_O_4_‐modified CeO_2_ strongly depends on the number of the Co_3_O_4_ surface sites accessible for the CO oxidation reaction, which is controlled by the route of introducing Co cations and by the Co loading. Considering the high activity of the presented catalysts in CO oxidation, with that of 16 wt % Co_3_O_4_−CeO_2_ approaching that of Pt/CeO_2_, it is anticipated that further optimization of the layered CeO_2_−Co_3_O_4_ nanocomposites may allow obtaining prototypes with even better low‐temperature TWC performance. In terms of a practical application in TWCs, the long‐term stability and activity in hydrocarbon oxidation and NO_*x*_ reduction in realistic exhaust gas feeds should also be investigated.

## Conflict of interest

The authors declare no conflict of interest.

## Supporting information

As a service to our authors and readers, this journal provides supporting information supplied by the authors. Such materials are peer reviewed and may be re‐organized for online delivery, but are not copy‐edited or typeset. Technical support issues arising from supporting information (other than missing files) should be addressed to the authors.

Supporting Information
