# Supplementary material for: Co3O4−CeO2 Nanocomposites for Low‐Temperature CO Oxidation
Source: Chemistry. 2021 Jun 10;27(68):16947–55. doi: 10.1002/chem.202100927 (PMC9292333; doi:10.1002/chem.202100927)
Supplement: Supplementary file 1 — Supporting Information [file CHEM-27-16947-s001.pdf]

Chemistry–A European Journal

Supporting Information

Co_3O_4–CeO_2 Nanocomposites for Low-Temperature CO Oxidation

Jingxia Yang, Nevzat Yigit, Jury Möller, and Günther Rupprechter*

Experimental Section

Materials

All solvents and solid chemicals were dried by standard methods, and all experiments involving metal alkoxides were carried out under moisture- and oxygen-free argon, using standard Schlenk or glove box techniques. Cerium *tert*-butoxide (CeB) (synthesized according to literature)[1] was used as cerium precursor while cobalt acetate tetrahydrate ($Co(OAc)_2·4H_2O$) was taken as a Co source. *p*-carboxybenzaldehyde oxime (POBC-H) was synthesized as described before.[2] The ligang acetaldoxime (AO) and the surfactant F127 were used as received. All solid chemicals were dried at $<10^{-3}$ mbar for at least 5 h to remove H_2O.

Synthesis of Co_3O_4 modified CeO_2 via different protocols (routes 1-3)

A combination of sol-gel and solvothermal methods was used to synthesize Co_3O_4 modified CeO_2 via three different routes, varying the way of adding the Co precursor. The atomic Co:Ce ratio was of 1:4 in all samples, leading to $Co_3O_4/(Co_3O_4+CeO_2)$ weight percentage of 10.4%. The chemical ratio of all precursor components was the same, namely CeB : $Co(OAc)_2$: oximate groups : F127 = 0.8 : 0.2 : 1.6 : 0.005. While the oximate groups for route **1** and **2** originated only from AO, the oximate groups for route **3** were acquired from both POBC and AO in a 1:3 ratio. The three prepared materials and their characterization by thermogravimetric analysis (TGA), N_2 sorption (BET surface area), X-ray diffraction (XRD), infrared spectroscopy (IR), small angle X-ray scattering (SAXS) and high resolution transmission electron microscopy (HR-TEM) were described in detail in [3] and are only briefly summarized in Table 1 of the main text.

Adjustment of the Co_3O_4 loading on CeO_2 via route 1

The molar percentage of $Co(OAc)_2/(CeB+ Co(OAc)_2)$ of Co was varied from 10% to 80% (corresponding to 4.9-65.1 wt. % of Co_3O_4 on CeO_2), with the materials synthesized by route **1**, as listed in Table 1. The total molar amount of CeB+$Co(OAc)_2$ is 5 mmol, and the molar ratio of the precursor components was CeB : $Co(OAc)_2$: AO : F127 = (5-x) : x : 2(5-x): 0.025 (x=0.5 for Co%=10%; x=1 for Co%=20%; x=1.5 for Co%=30%; x=4 for Co%=80%). Typically, (5-x) mmol CeB was mixed with 2(5-x) mmol of AO and 0.025 mmol of F127 in 20 ml of DME. The sol was treated as described in route 1 to get a Ce gel. Then, the gel was put into an autoclave with 30 mL EtOH and x mmol $Co(OAc)_2$ and treated solvothermally at 200°C for 6 h. After filtration, washing and drying, the samples are labeled as “10% Co **1**-STE” (**S**olvothermal **T**reatment in **E**thanol), ‘20% Co **1**-STE’, ‘30% Co **1**-STE’ and ‘80% Co **1**-STE’. Part of each sample was further **A**ir-**C**alcined (AC) at 500°C for 2 h (ramping rate: 2°C/min) and is labeled ‘**1**-STE-AC’.

Table 1 Preparation of Co_3O_4-CeO_2 nanocomposites with different loading.

Chemicals / Sample	CeB (mmol)	$Co(OAc)_2$ (mmol)	Co/(Ce+Co) (mol%)	$Co_3O_4/(Co_3O_4+CeO_2)$ (wt%)
10% Co **1**-STE	4.5	0.5	10	4.9
20% Co **1**-STE	4	1	20	10.4
30% Co **1**-STE	3.5	1.5	30	16.6
80% Co **1**-STE	1	4	80	65.1

Reference catalysts of CeO_2, Co_3O_4 and Pt/CeO_2

The reference CeO_2 was synthesized using route 1 without adding $Co(OAc)_2$ in the solvothermal step. Commercial Co_3O_4 from Fluka (purity 99.5%) was used as received. A reference catalyst of 1 wt.% Pt/CeO_2 was prepared by impregnation. First, 0.02 g of Platinum(II) bis(acetylacetonate) were dissolved in 10 mL toluene and mixed with 0.99 g of commercial CeO_2 dispersed in 40 mL toluene. After 2 h of stirring at room temperature, the entire solution was evaporated to dryness by gradually increasing temperature to 100 °C. The dried solid was subsequently calcined at 300 °C for 2 h in air. Pretreatment was carried out by oxidation in 20 vol.% O_2 in He at 400 °C (30 min), followed by reduction in 5 vol. % H_2 in He at 200 °C (30 min). This yielded Pt nanoparticles of 1.7 nm mean size, as revealed by CO chemisorption.

Characterization

Temperature programmed reduction

CO temperature-programmed reduction (CO-TPR) was performed in a continuous-flow fixed-bed quartz reactor under atmospheric pressure. Catalyst samples (20 mg) were pre-treated in 50 ml/min synthetic air (200°C for 30 min by 10°C/ min ramping rate). After cooling to room temperature, the samples were exposed to 5 vol% CO in He (total flow 50 ml/min), followed by heating to 800°C (10°C/min). The gas stream was analyzed by an online quadrupole mass spectrometer (QMS) (Prisma Plus QMG 220, Pfeiffer Vacuum) equipped with a Faraday detector.

Catalytic performance

The CO oxidation reaction was performed by using 10 mg of the samples or reference catalysts in the same type of quartz reactor. The samples were pretreated with synthetic air (50 ml/min) at 200°C for STE samples or at 400°C for STE-AC and oxide reference catalysts for 30 min (heating rate 10°C/min). For Pt/CeO_2, after oxidation at 400°C, the sample was reduced at 200 °C for 30 min (heating rate 10°C/min) with 5 vol.% H_2 in He. After cooling to 30°C, 5 vol% CO, 10 vol% O_2 and 85 vol% He mixture (total flow 50 ml/min) were introduced, and the system was heated up to 400°C (ramping rate of 2.5°C/min). The concentrations of CO and CO_2 in the outlet streams were monitored by gas chromatography with a HP-PLOT Q column and a flame ionization detector (FID).

Structure and surface characterization

XRD measurements were performed on a Philips X'Pert diffractometer using Cu-K_α radiation (λ=1.5406 Å; X-ray tube at 40 kV and 40 mA) operating in Bragg-Brentano reflection geometry. The scanning range was 5-80° (2θ) in step scan mode of 0.05° (2θ), with 4.5 s per step.

Nitrogen sorption measurements were performed on an ASAP 2020 (Micromeritics). The samples were degassed in vacuum at 80°C for at least 5 h prior to measurement. The total surface area was calculated according to Brunauer, Emmett and Teller (BET) and the pore size distribution (from the desorption branch) according to Barrett, Joyner and Halenda (BJH).

The Pt dispersion and mean particle size were determined via CO-chemisorption, assuming a CO:Pt ratio of 1:1.

References

[1] a) P. S. Gradeff, F. G. Schreiber, K. C. Brooks, R. E. Sievers, *Inorg. Chem.* **1985**, *24*, 1110-1111; b) W. J. Evans, T. J. Deming, J. M. Olofson, J. W. Ziller, *Inorg. Chem.* **1989**, *28*, 4027-4034.

[2] a) J. Yang, J. Akbarzadeh, C. Maurer, H. Peterlik, U. Schubert, *J. Mater. Chem.* **2012**, *22*, 24034-24041; b) J. Yang, M. Puchberger, R. Qian, C. Maurer, U. Schubert, *Eur. J. Inorg. Chem.* **2012**, *2012*, 4294-4300.

[3] J. Yang, L. Lukashuk, J. Akbarzadeh, M. Stoeger-Pollach, H. Peterlik, K. Foettinger, G. Rupprechter, U. Schubert, *Chem. – Eur. J.* **2015**, *21*, 885 - 892.
